# Effect of Chloride Passivation on Recombination Dynamics in CdTe Colloidal Quantum Dots

**DOI:** 10.1002/cphc.201402753

**Published:** 2015-01-14

**Authors:** Daniel Espinobarro-Velazquez, Marina A Leontiadou, Robert C Page, Marco Califano, Paul O'Brien, David J Binks

**Affiliations:** [a] School of Physics and Astronomy and Photon Science Institute, University of Manchester Oxford Road, Manchester M13 9PL (UK) E-mail: david.binks@manchester.ac.uk; [b] School of Chemistry, University of Manchester Oxford Road, Manchester M13 9PL (UK); [c] Institute of Microwaves and Photonics, School of Electronic and Electrical Engineering, University of Leeds Woodhouse Lane, Leeds LS2 9JT (UK)

**Keywords:** chloride, nanoparticles, passivation, photoluminescence, quantum dots

## Abstract

Colloidal quantum dots (CQDs) can be used in conjunction with organic charge-transporting layers to produce light-emitting diodes, solar cells and other devices. The efficacy of CQDs in these applications is reduced by the non-radiative recombination associated with surface traps. Here we investigate the effect on the recombination dynamics in CdTe CQDs of the passivation of these surface traps by chloride ions. Radiative recombination dominates in these passivated CQDs, with the radiative lifetime scaling linearly with CQD volume over *τ*_r_=20–55 ns. Before chloride passivation or after exposure to air, two non-radiative components are also observed in the recombination transients, with sample-dependent lifetimes typically of less than 1 ns and a few ns. The non-radiative dynamics can be explained by Auger-mediated trapping of holes and the lifetimes of this process calculated by an atomistic model are in agreement with experimental values if assuming surface oxidation of the CQDs.

## 1. Introduction

Colloidal quantum dots (CQDs) have applications as the light emitting or absorbing species in a range of optoelectronic devices based on organic charge transporting layers, including light emitting diodes,[1] photovoltaic cells,[2–4] photodetectors,[3, 5] holographic data stores and image processors.[6] CQDs are well-suited for use in combination with organic materials because they can also be synthesised and processed by cost-effective and scalable solution-based methods. Moreover, their emission wavelength and absorption edge is size-tunable, enabling facile optimisation for particular device designs, and they can also benefit from high photoluminescence (PL) quantum yield (QY) and narrow band emission.[7, 8]

However, the small size (typically 2–5 nm) and consequently large surface-area-to-volume ratio of CQDs can result in a high concentration of trap states associated with unsaturated bonds on the surface. These surface traps can enable non-radiative recombination pathways that compromise device efficiency by reducing the QY of radiative recombination or charge extraction, or by decreasing charge mobility.[9, 10] Suppression of unwanted surface-related recombination is thus essential to maximising the performance of devices incorporating CQDs.[2, 11–13]

CQD surface traps have previously been passivated either by organic ligands or by a wide-band-gap semiconductor shell.[14, 15] However, both of these techniques have their drawbacks. Ligand coverage of the surface has proved to be incomplete,[16, 17] leaving some traps unpassivated, but introducing a barrier to charge transfer. The wide-band shell can prevent charges photogenerated within the CQD interacting with surface traps, resulting in nearly 100 % PLQY if the CQD-shell is grown without defects[18–20] but inhibiting charge transport.[21] Passivation of surface traps with halide ions has recently emerged[12, 22] as a promising method to significantly improve device performance, resulting, for instance, in an increase to nearly 9 % in the record efficiency for a CQD-based solar cell.[23] These impressive results have been attributed to the ability of compact halide ions to passivate sites on the CQD surface that are inaccessible to bulkier ligands, and to do so without introducing a charge transport barrier.[12] Halide passivation also greatly reduces the sensitivity of CQDs to air exposure, by binding to sites on the CQD’s surface otherwise subject to oxidative attack, allowing device fabrication in ambient conditions and thus reducing potential production costs.[24]

Until now, the PLQY values reported for halide-passivated CQDs, which depend on the ratio of the rates of radiative and non-radiative recombination, have all been significantly less than unity indicating that the complete passivation of surface traps has still not been achieved, despite the impressive improvements in device performance. However, we have recently developed a technique that uses chloride ions to passivate CdTe CQDs that can result in near-unity PLQY that is, almost complete surface passivation.[25] In this work, we report a study of the effects of chloride ion passivation on recombination dynamics in these CQDs. The simplified recombination transient in the passivated CQDs allows the underlying charge dynamics to be studied free of the sample-specific influence of trapping, which hitherto has complicated analysis. These results are also compared with the dynamics of unpassivated CQDs and with those exposed to air, enabling the study of the dynamics of the trapping process itself. Recent work has shown that the Auger-mediated trapping (AMT) of holes can be used to explain the charge dynamics observed in a number of CQD types, including CdSe and InAs/ZnSe CQDs.[26] An atomistic model of a CdTe CQD has been developed and is used to calculate AMT rates for holes, and the results are compared to the non-radiative lifetimes found experimentally.

## 2. Results and Discussion

### 2.1. Radiative Recombination

**Figure 1:**
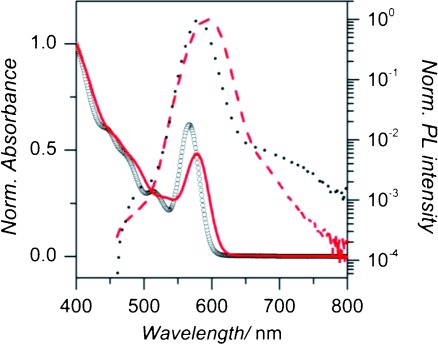
Normalised absorption spectra for an untreated (circles) and a treated (solid line) CdTe CQD sample. Also shown are the PL spectra for the same untreated (dots) and treated (dashed line) samples. For details of the samples, see #4 and #4^+^ in Table S1 in the Supporting Information.

Representative normalised absorption and PL spectra for a sample of CQDs before and after the surface passivation treatment are shown in Figure 1. The spectra are largely unchanged by the treatment process, except for a red-shift of about *λ*=10 nm. This spectral shift on passivation has previously been shown to depend on the treatment time and the concentration of chloride ions used.[25] Moreover, the addition of chloride ions to the CdTe surface was shown to fill states near to the valance band maximum, narrowing the band gap and thus red-shifting the emission peak and absorption edge.[22] Notably, the chloride treatment produces a dramatic increase in PLQY, from typically about 5 % before treatment to greater than 90 % in some cases afterwards. The position of the PL and absorbance peaks, and the QY values for all the samples used in this study, before and after passivation, are given in the Supporting Information (see Table S1 in S1).

**Figure 2:**
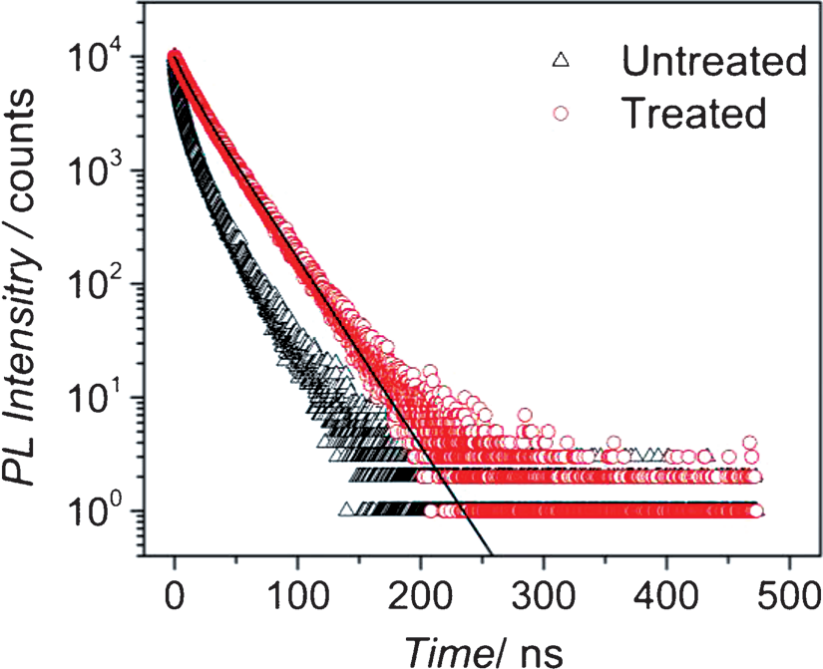
Representative transient PL decay, *I*_PL_(*t*), showing multi-exponential decay for a CdTe CQD sample before chloride treatment (triangles) and mono-exponential decay for the same sample after treatment (circles). See also Figures S1 and S3 in the Supporting Information.

As noted previously,[25] the dominance of radiative recombination in passivated CQDs, as evidenced by the near unity PLQY, also affects the form of the PL decay transients, *I*_PL_(*t*). As shown in Figure 2 for a typical sample (and in the Supporting Information for the other samples reported here, see Figure S1), before passivation *I*_PL_(*t*) is multi-exponential in form. In contrast, after passivation the transient is well-described by a mono-exponential decay, and hence can be characterised by a single time constant, *τ*_PL_. This mono-exponential form was found to be independent of excitation power over nearly 2 orders of magnitude (see Figure S2).

**Figure 3:**
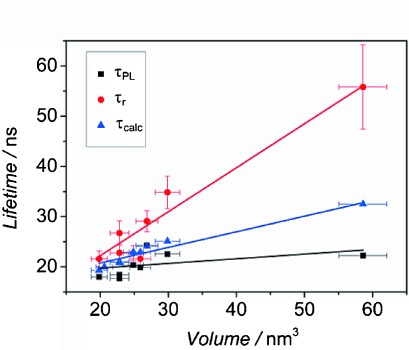
PL, *τ*_PL_ (squares), radiative, *τ*_r_ (circles) and calculated, *τ*_calc_, (triangles) lifetimes as a function of CQD volume for a range of samples (see treated samples #1–7 in Table S1). Linear fits are shown as solid lines.

The form of *I*_PL_(*t*)for passivated samples allows the radiative lifetime, to be estimated as *τ*_r_≈*τ*_PL_/QY, a procedure only possible if the transient is mono-exponential. PL intermittency (“blinking”), which can also result from trapping,[27] decreases the number of CQDs that contribute to the PL decay, reducing QY but leaving *τ*_PL_ unaffected. Similarly, trapping of hot excitons[28] also reduces the QY without affecting the rate of band-edge recombination. Thus, the true radiative lifetime lies between *τ*_PL_ and the value estimated from *τ*_r_≈*τ*_PL_/QY, with the accuracy of the estimate improving for high QY, as for the samples studied here. Figure 3 shows the values of *τ*_r_, which range between 20 ns and 55 ns, found in this way for transients at the PL peak for a number of different samples, as a function of CQD volume. The diameter of each CQD was determined from the position of the absorption edge, using a previously reported empirical relationship,[29] and the volume calculated assuming a spherical shape. As expected for radiative decay,[30] *τ*_r_ scales linearly with CQD volume. In comparison, previous studies^[31, 32]^ have reported radiative lifetimes ranging between *τ*_r_=20 ns and *τ*_r_=40 ns for CdTe CQDs of similar sizes. However, these works directly associated the radiative lifetime with the observed PL decay constant that is, are more comparable with the values of *τ*_PL_ found here. Thus, also shown in Figure 3 are *τ*_PL_ and the PL lifetimes, *τ*_calc_, calculated from the empirical relationship between the recombination rate and the peak PL photon energy reported by de Mello Donega et al.[32] The values of *τ*_PL_ and *τ*_calc_ are broadly comparable, whereas the values of *τ*_r_ are systematically longer than either *τ*_PL_ or *τ*_calc_, consistent with *τ*_r_≈*τ*_PL_/QY representing the upper limit for radiative lifetime.

**Figure 4:**
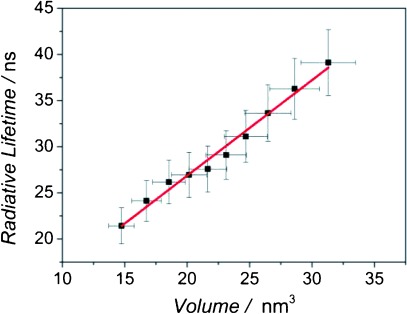
Radiative lifetime, *τ*_r_, as a function of CQD volume within the size distribution of a sample. A linear fit is shown by the red line.

The PL peak is widened by the size dispersion in a sample, with smaller than average CQDs contributing to the short wavelength side of the peak and larger CQDs to the long wavelength side. Thus, measuring *I*_PL_(*t*) at different wavelengths across the PL peak allows different diameters of CQDs to be studied, within the overall size distribution of the sample. As shown in the Supporting Information (see Figure S4), a progressive slowing of the PL decay is seen for longer wavelengths within the PL peak. The diameter of the CQDs corresponding to each of these decays can also be calculated from the PL wavelength used, taking into account the Stokes shift between PL peak and absorption edge. Assuming a constant QY value for all CQD diameters, the values of *τ*_r_ extracted at different wavelengths across the PL peak were extracted and are shown in Figure 4. Here, *τ*_r_ also scales linearly with CQD volume showing that the observed change in decay transient across the PL is due to the different CQD diameters sampled at different wavelengths.

### 2.2. Non-radiative Recombination

**Figure 5:**
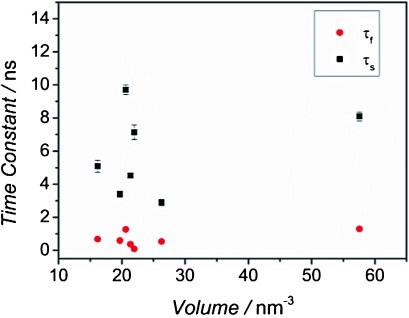
Fast and slow time constants for different unpassivated samples in respect to the volume.

*I*_PL_(*t*) observed for the untreated samples could be well-described by tri-exponential of the form [Eq. (1)]:


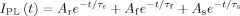
(1)

in which *τ*_r_ was fixed to the value extracted for the same sample after chloride passivation; *τ*_f_ and *τ*_s_ are time constants associated with additional fast and slow non-radiative components, respectively; and *A*_r_, *A*_f_ and *A*_s_ are the amplitudes associated with the radiative, fast non-radiative and slow non-radiative components, respectively. The values of *τ*_f_ and *τ*_s_ found from a fit to *I*_PL_(*t*) for a number of samples of different CQD diameters are shown in Figure 5. In this case, there is no systematic variation with CQD volume, confirming that these time constants are not associated with radiative recombination. As discussed in Section 2.1, the true value of the radiative lifetime will lie between *τ*_r_≈*τ*_PL_/QY and *τ*_PL_, and so to check the sensitivity to the value used for radiative lifetime, the tri-exponential fitting procedure was repeated with the lifetime of the first component fixed to *τ*_PL_ rather than *τ*_r_. The resulting values of *τ*_f_ and *τ*_s_ (see Figure S5) are similar though reduced. However, a systematic variation with CQD volume is now evident, indicating that the values of *τ*_f_ and *τ*_s_ are somewhat influenced by radiative recombination if *τ*_PL_ is used for the first component results. That is, there is a less reliable separation of the radiative and non-radiative dynamics.

### 2.3. Effect of Exposure to Air

**Figure 6:**
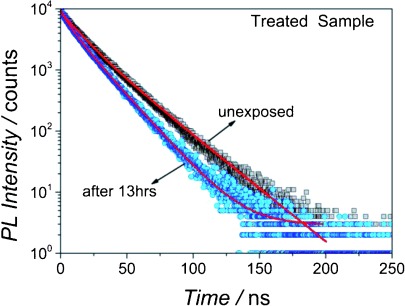
Representative PL transient decays for a chloride-treated sample before and after *t*=13 h exposure to air (see details of sample #7^+^ in the Supporting Information).

**Figure 7:**
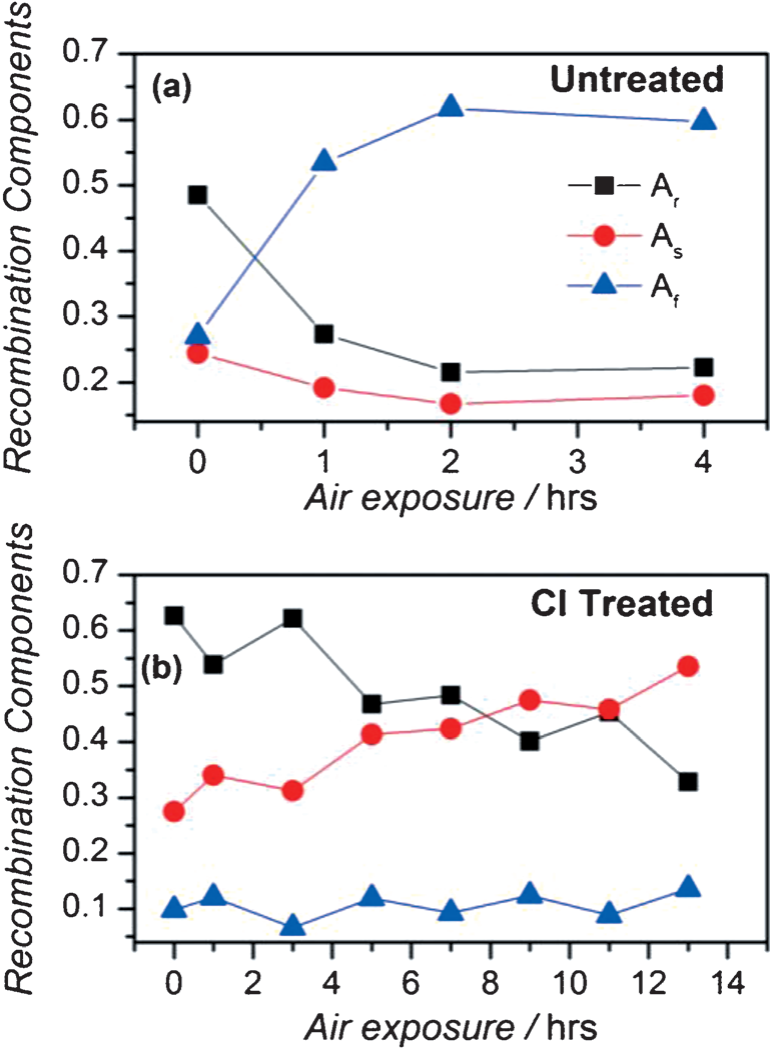
Change in the radiative (*A*_r_), slow (*A*_s_) and fast (*A*_f_) non-radiative recombination components with air exposure a) before and b) after Cl treatment (see samples #7 and #7^+^ in the Supporting Information).

*I*_PL_(*t*) changes significantly for both chloride-treated and untreated samples on exposure to air, in parallel with a decrease in QY,[25] indicating that non-radiative pathways are produced or altered by oxidation. As shown in Figure 6, *I*_PL_(*t*) for a passivated sample becomes multi-exponential in form over a number of hours after first contact with the air, and can now be well-described by Eq. (1), by using the value of *τ*_r_ found from the transient obtained before exposure to air. For increasing air exposure, the time constants remain constant but the amplitudes of the non-radiative components increase at the expense of *A*_r_, consistent with the diminishing QY, with *A*_s_ showing the greatest increase. For the untreated sample *A*_r_ also decreases with exposure to air, again consistent with the reducing QY, but in this case only *A*_f_ increases with *A*_s_ reducing somewhat with oxidation time. The behavior of the fractional contribution of each component is shown in Figure 7 for both the case of untreated and chloride-treated CQDs.

## 3. Auger-mediated Trapping Model

**Figure 8:**
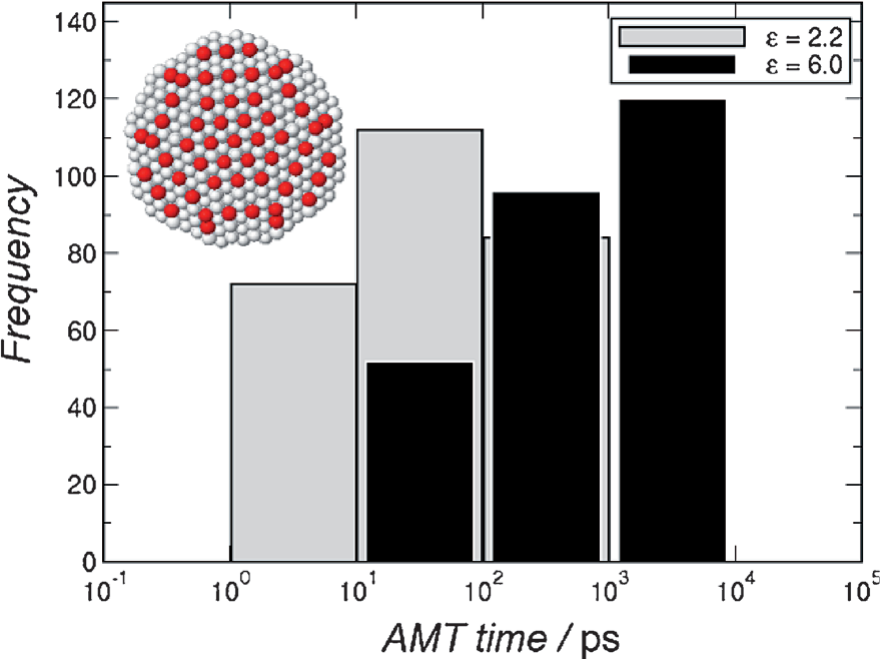
Distribution of hole AMT times to unsaturated Te bonds located on the surface of a *R*=2.3 nm CdTe core, calculated for different dielectric environments: common solvents (*ε*=2.2, grey bars) and CdO (*ε*=6, black bars). The width of the black bars was reduced for clarity. The inset represents an accurate map of the positions of surface Te atoms with unsaturated bonds (dark spheres). Cd atoms and 4-coordinated Te atoms are displayed in white for clarity.

If a photogenerated hole undergoes a (trapping) transition to a state in the gap, its excess energy can be transferred non-radiatively to the electron, exciting it to a state above the conduction band edge. This AMT process, has been used recently[26, 33] to explain many features observed experimentally in the charge dynamics of CQDs made of different materials in different environments. Here we extend its application to investigate PL dynamics in CdTe dots. AMT rates for the transition from the band edge Exciton, |*e*_CBM_,*h*_VBM_>, to a surface-trapped Exciton, |*e*_n_,*h*_trap_> (in which *e*_n_ is an excited electron state and *h*_trap_ a surface hole trap state) were calculated in CdTe CQDs with *R*=2.3 nm, by using standard time-dependent perturbation theory[34] and LDA-quality wave functions obtained within the atomistic semiempirical pseudopotential method, (further details on the method can be found in Refs. [26, 33]), for trap states—Te unsaturated bonds—located at different positions on the surface. As in the case of CdSe,[26, 33] the calculated AMT times were found to depend on the number of dangling bonds of each surface Te atom: for dots dispersed in common solvents (such as toluene), traps obtained from atoms with single dangling bonds were generally less efficient (i.e. had a slower trapping time) than those created from atoms with double dangling bonds.[35] Unlike the case of CdSe, though, the distribution of the trapping times was narrow, ranging from a few picoseconds to a few hundreds of picoseconds (see the green bars in Figure 8), as it was found for InAs[26, 33] (which has the same crystal structure as CdTe). This result is qualitatively consistent with our experimental observation of a slow and a fast non-radiative component in the PL decay.

The value of the trapping times depends on the dielectric environment of the trap. The above calculations assumed that the dots were embedded in a matrix with a relative dielectric constant of *ε*=2.2, which corresponds to the toluene used as a solvent. For a value of *ε*=6, however, AMT becomes less efficient for all traps, with the transfer times to the efficient traps increasing by about 3 orders of magnitude, from a few picoseconds to a few nanoseconds (see the black bars in Figure 8), that is, in broad quantitative agreement with the experimentally measured time constants for non-radiative recombination. According to ab initio many-body calculations[36] the dielectric constant for cadmium oxide (CdO) has this value, which, coupled with the increasing contribution of non-radiative recombination for greater exposure to air noted above, indicates that oxidation of the CQD surface plays a key role in producing the nanosecond time scale non-radiative recombination observed. The tetradecylphosphonic acid used to passivate the untreated CQDs also bonds Cd atoms to oxygen, which, given that the same time constants describe *I*_PL_(*t*) before and after exposure to air, suggests that this too produces a dielectric environment resulting in nanosecond time scale non-radiative recombination. The relative contributions of the fast and slow non-radiative components, and their evolution upon exposure to air, detailed in Sections 2.2 and 2.3, reflect the effect on the distribution of trapping times of the different ligands and chloride ions on both the passivation of dangling bonds and on the local dielectric environment.

## 4. Summary and Conclusions

The effect of chloride ion passivation on the recombination dynamics in CdTe CQDs was studied. The almost complete passivation of the chloride-treated CQDs produced a mono-exponential photoluminescence decay transient that enabled the radiative lifetime to be determined free of the sample-specific contribution of surface trapping, which was found to scale linearly with CQD volume as expected. Before the chloride treatment and after exposure to air, the decay transient could be well-described by a tri-exponential, characterised by the radiative lifetime and fast and slow time constants associated with non-radiative recombination. The contribution of these non-radiative components grew on exposure to air for both the treated and untreated samples.

These observations are consistent with a model of non-radiative recombination based on the AMT of holes by surface dangling bonds. Calculations of trapping times by using an atomistic semiempirical pseudopotential method demonstrated that the there are two types of holes traps, corresponding to Te atoms with either one or two dangling bonds. Each trap type produced a distribution of trapping times depending on the location of the Te atom on the CQD surface. The values of the trapping times depended on the dielectric environment of the trap. For a dielectric constant corresponding to the solvent used in the experiments, the trapping times were in the picosecond time scale. However, using a dielectric constant corresponding to CdO produced trapping times on the nanosecond scale, in agreement with experiment. This suggests that oxidation not only produces traps, as evidenced by the increasing non-radiative contribution to the decay transient on exposure to air, but also determines the time scale of non-radiative recombination.

## Experimental Section

The CdTe CQDs were synthesised by using a previously published method[18] and well-controlled growth times to produce different diameters, capped with tetradecylphosphonic acid and trioctylphosphine ligands. The chloride treatment has been described in detail recently[25] and the same procedure was followed here. The concentration of chloride used in the treatment was equivalent to a density on the CQD surface of 96 ions/nm^2^, and it has been shown[25] that a similar density of oleylamine ligands was also present on the surface. All treated and untreated samples were diluted in toluene and placed in quartz cuvettes (always sealed under N_2_ atmosphere) for further investigation. The samples were stable, with unchanging QY, if kept under an inert atmosphere.[25]

PL transients were recorded by using time correlated single photon counting. The pump beam was from a mode-locked Ti:sapphire laser (Mai Tai HP, Spectra-Physics), providing *t*=100 fs pulses at a repetition rate of 80 MHz and a wavelength of *λ*=820 nm. With the use of an acousto-optic pulse picker (Pulse Select, APE) the rate was reduced to 2 MHz, and then the wavelength was converted to *λ*=410 nm through second harmonic generation (APE Harmonic Generator). After excitation the PL emission was directed into a monochromator (Spex 1870c) and detected at the PL peak (or tuned at the preferred wavelength within the range of the PL spectra of each sample) by a multi-channel plate (Hamamatsu R3809U-50). The time correlation of the detected photons was performed by using a PC card (TCC900, Edinburgh Instruments).

The PL emission spectra and PLQY for the CQD samples were measured by using a spectrofluorometer (FluoroLog 33-22iHR, Jobin–Yvon) with a built-in integrating sphere (F-3018, Jobin–Yvon). The excitation wavelength was set to *λ*=450 nm with a bandwidth of 1.3 nm. Absorbance spectra were obtained by using a PerkinElmer Lambda-1050 spectrophotometer.

## Supporting Information

As a service to our authors and readers, this journal provides supporting information supplied by the authors. Such materials are peer reviewed and may be re-organized for online delivery, but are not copy-edited or typeset. Technical support issues arising from supporting information (other than missing files) should be addressed to the authors.

miscellaneous_information
