# Supplementary material for: Effect of Chloride Passivation on Recombination Dynamics in CdTe Colloidal Quantum Dots
Source: Chemphyschem. 2015 Jan 14;16(6):1239–44. doi: 10.1002/cphc.201402753 (PMC4501323; doi:10.1002/cphc.201402753)
Supplement: Supplementary file 1 — miscellaneous_information [file cphc0016-1239-sd1.pdf]

CHEMPHYSCHEM

Supporting Information

# Effect of Chloride Passivation on Recombination Dynamics in CdTe Colloidal Quantum Dots

Daniel Espinobarro-Velazquez,[a] Marina A. Leontiadou,[a] Robert C. Page,[b] Marco Califano,[c] Paul O'Brien,[b] and David J. Binks*[a]

cphc_201402753_sm_miscellaneous_information.pdf

## S1. Transient PL decay traces

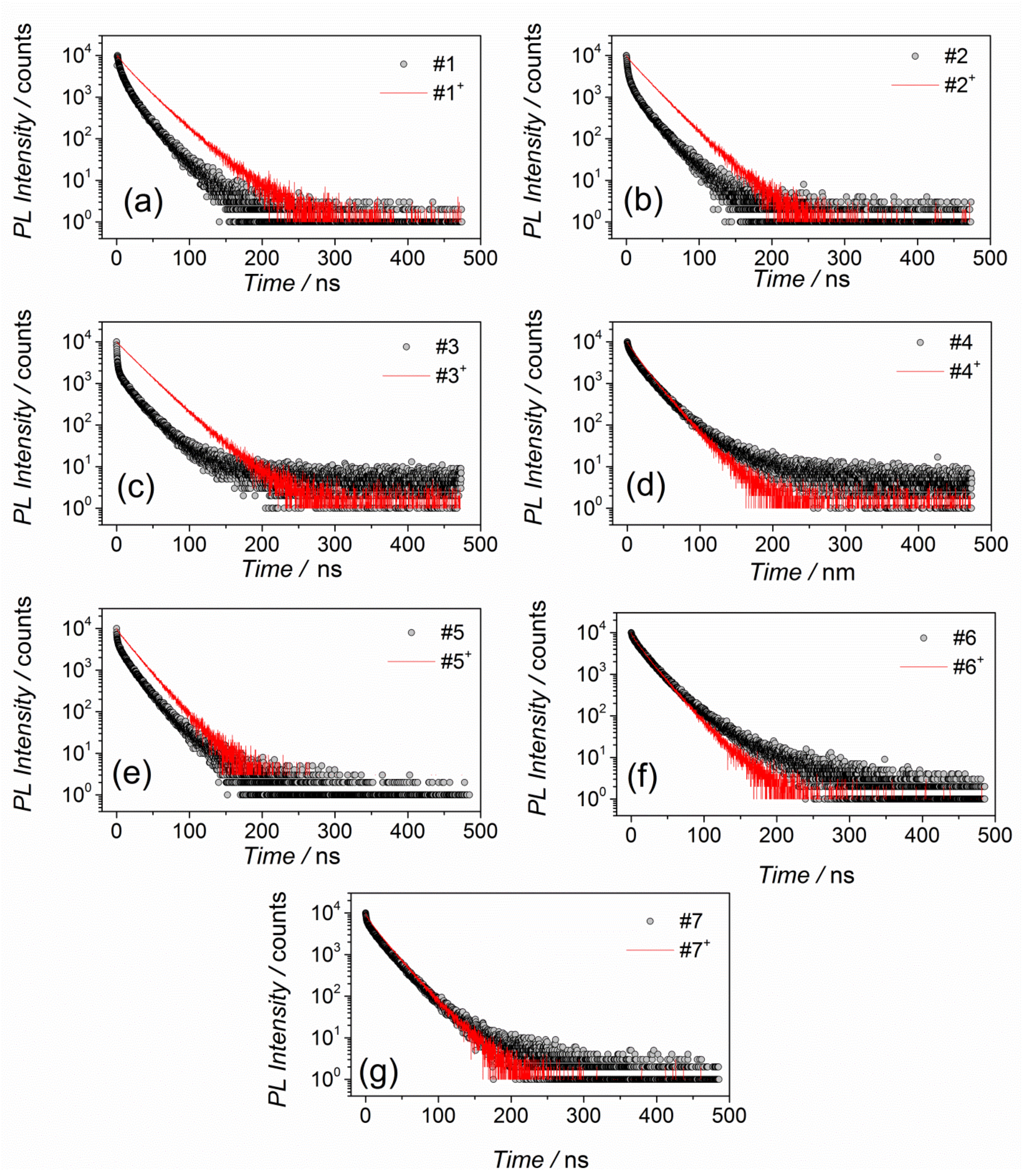


**Figure S1. (a)-(g)**Transient PL decays, $I_{PL}(t)$, showing multi-exponential decay for a CdTe CQD sample before chloride treatment (circles) and mono-exponential decay for the same sample after treatment (solid lines). Samples parameters are given in Table S1.

## S2. Sample properties.

**Table S1.** Sample parameters.

| Sample | Diameter[a] / ±0.1 nm | Abs peak / nm | PL peak /nm | PLQY / ±6 % |
|---|---|---|---|---|
| **#1** | 4.8 | 668 | 678 | 3 |
| **#1+** | 4.8 | 669 | 677 | 40 |
| **#2** | 3.9 | 620 | 631 | 1 |
| **#2+** | 3.8 | 619 | 630 | 65 |
| **#3** | 3.7 | 603 | 613 | 4 |
| **#3+** | 3.7 | 606 | 622 | 83 |
| **#4** | 3.4 | 567 | 580 | 4 |
| **#4+** | 3.5 | 582 | 593 | 66 |
| **#5** | 3.4 | 572 | 577 | 5 |
| **#5+** | 3.7 | 601 | 612 | 92 |
| **#6** | 3.1 | 541 | 557 | 8 |
| **#6+** | 3.4 | 562 | 577 | 80 |
| **#7** | 3.5 | 576 | 586 | 15 |
| **#7+** | 3.5 | 582 | 595 | 81 |

[+] Chloride treated samples, [a] Determined using empirical relationship of Yu et al[1]

## S3. Independence of PL decay traces on excitation power.

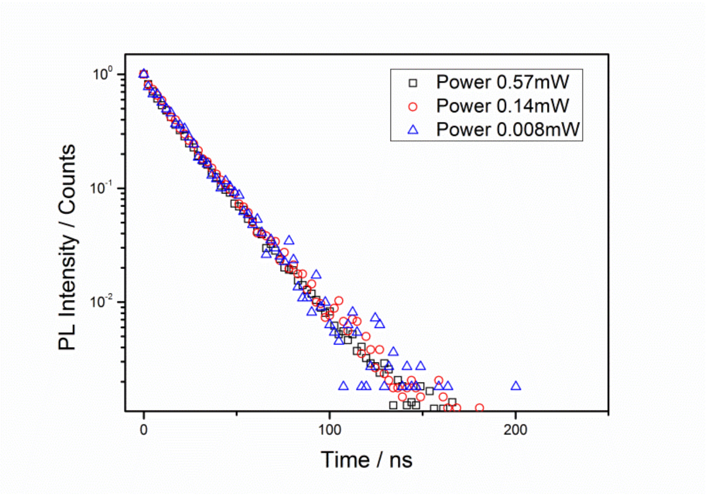


**Figure S2.** Comparison of decay traces for sample #4+ (see Table S1 above) produced by a range of average excitation powers. The pulse repetition rate was 2 MHz and the pump beam was focused to a spot size of ~3 µm within the sample using a microscope objective (Olympus, DPLAN 10).

## S4. Example PL decay trace plotted on a linear scale.

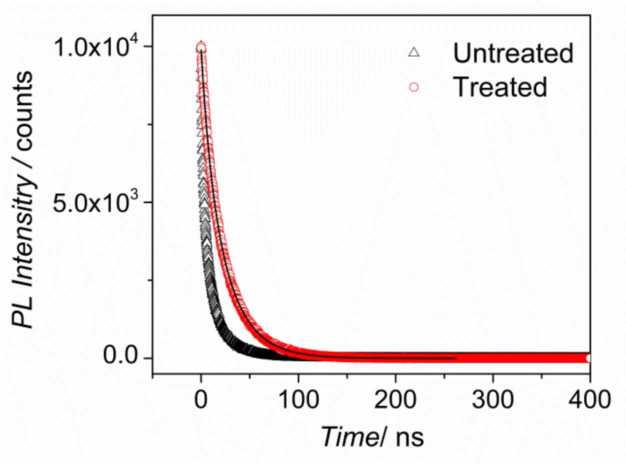


**Figure S3.** Decay transients for sample #1 presented on a linear scale.

## S5. PL decay traces at different wavelengths across the PL spectrum.

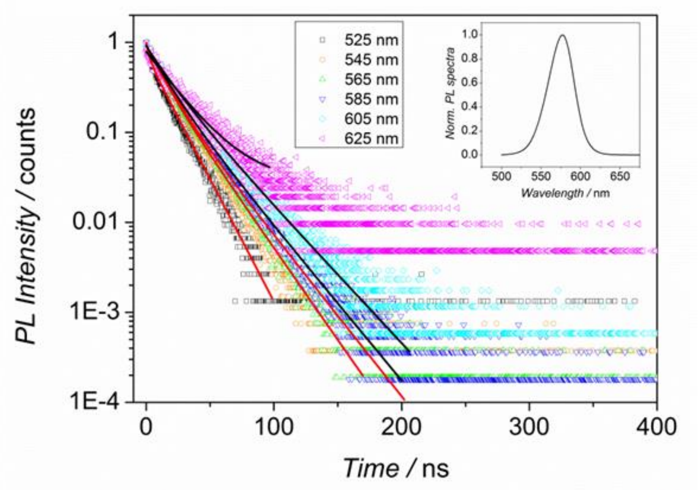


**Figure S4.** Decay traces for treated sample #4$^+$ at different wavelengths across the PL spectra (see Inset). Solid lines are mono-exponential fittings. Inset; the normalized PL spectra for sample #4$^+$.

## S6 Fast and slow non-radiative time constants

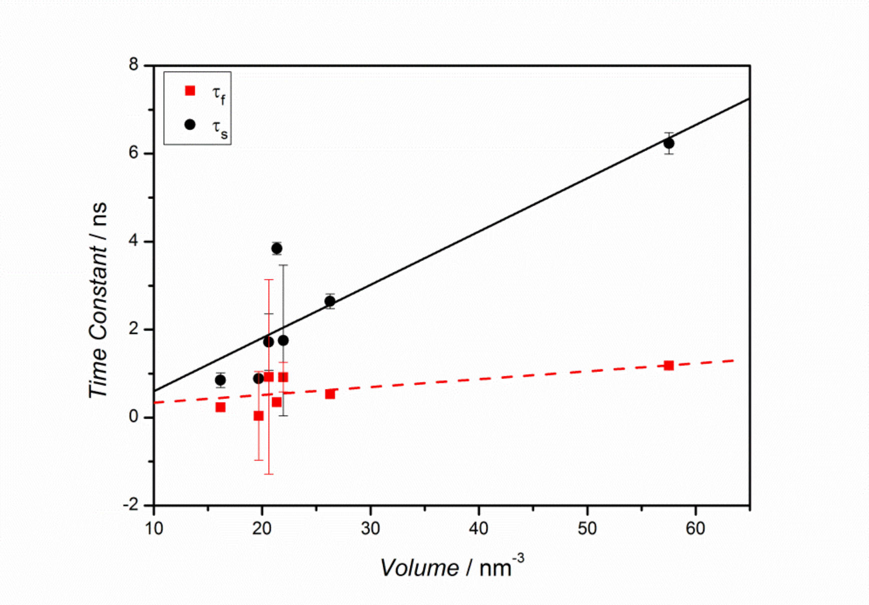


**Figure S5. The fast and slow non-radiative time constants, .** $\tau_f$ and $\tau_s$, **for different CQD sizes found by fixing the first time constant to** $\tau_{PL}$.

## Reference.


[1] a) W. W. Yu, L. H. Qu, W. Z. Guo, X. G. Peng, *Chem Mater* **2004**, *16*, 560-560; b) W. W. Yu, L. H. Qu, W. Z. Guo, X. G. Peng, *Chem Mater* **2003**, *15*, 2854-2860.
